# Eimeria tenella ROP kinase EtROP1 induces G0/G1 cell cycle arrest and inhibits host cell apoptosis

**DOI:** 10.1111/cmi.13027

**Published:** 2019-04-24

**Authors:** Mamadou Amadou Diallo, Alix Sausset, Audrey Gnahoui‐David, Adeline Ribeiro E. Silva, Aurélien Brionne, Yves Le Vern, Françoise I. Bussière, Julie Tottey, Sonia Lacroix‐Lamandé, Fabrice Laurent, Anne Silvestre

**Affiliations:** ^1^ Infectiologie et Santé Publique, INRA Université de Tours Nouzilly France; ^2^ BOA, INRA Université de Tours Nouzilly France

**Keywords:** apicomplex, *Eimeria*, p53, pseudokinase, ROP kinase, *Toxoplasma*

## Abstract

Coccidia are obligate intracellular protozoan parasites responsible for human and veterinary diseases. *Eimeria tenella*, the aetiologic agent of caecal coccidiosis, is a major pathogen of chickens. In *Toxoplasma gondii*, some kinases from the rhoptry compartment (ROP) are key virulence factors. ROP kinases hijack and modulate many cellular functions and pathways, allowing *T. gondii* survival and development. E. tenella's kinome comprises 28 putative members of the ROP kinase family; most of them are predicted, as pseudokinases and their functions have never been characterised. One of the predicted kinase, EtROP1, was identified in the rhoptry proteome of E. tenella sporozoites. Here, we demonstrated that EtROP1 is active, and the N‐terminal extension is necessary for its catalytic kinase activity. Ectopic expression of EtROP1 followed by co‐immunoprecipitation identified cellular p53 as EtROP1 partner. Further characterisation confirmed the interaction and the phosphorylation of p53 by EtROP1. E. tenella infection or overexpression of EtROP1 resulted both in inhibition of host cell apoptosis and G0/G1 cell cycle arrest. This work functionally described the first ROP kinase from E. tenella and its noncanonical structure. Our study provides the first mechanistic insight into host cell apoptosis inhibition by E. tenella. EtROP1 appears as a new candidate for coccidiosis control.

## 1 INTRODUCTION

Apicomplexa are protozoan parasites that cause significant human diseases (*Plasmodium*, *Toxoplasma*, *Cryptosporidium*) and are of major agricultural importance (*Cryptosporidium*, *Eimeria*, *Neospora*, *Theileria*). *Eimeria tenella*, one of the most virulent species that causes avian coccidiosis, is responsible for worldwide economic losses in poultry industry. To develop efficient and sustainable control strategies of coccidiosis, it is a priority to identify new therapeutic targets or virulence factors expressed by E. tenella. In this context, we focused on kinases from E. tenella that may represent interesting targets for anticoccidial control.

Apicomplexan kinome comprises members from eukaryotic protein kinases (ePKs) (Doerig et al., 2008) and also some specific kinase families such as FIKKs (*Plasmodium*, *Theileria*, *Babesia*, …) or ROP kinases (ROPKs) for coccidia (*Toxoplasma*, *Eimeria*, *Neospora*; Talevich, Mirza, & Kannan, 2011). The ROPK family has been under positive selection since coccidia diverged. ROPK sequences are highly divergent from ePK sequences (Peixoto et al., 2010). Genomic analyses have shown that E. tenella encodes 90 PKs among which 28 likely belong to the ROP kinase family and that most of them are putative pseudokinases because of the absence of a complete catalytic domain (Peixoto et al., 2010; Reid et al., 2014). By comparing ROPK allelic sequences between three strains of *Toxoplasma gondii* corresponding to three classes of virulence (GT1, type I, highly virulent; ME49, type II, moderately virulent; VEG, type III, non‐virulent), it was demonstrated that several ROPKs were highly polymorphic pathogenicity factors. The individual deletion of ROPK gene in a type II *T. gondii* resulted in less virulent strains for 16 ROPK genes (Fox et al., 2016). Several *T. gondii* ROPKs are involved in host cell reprogramming. For instance, TgROP18, responsible for inactivation of the host defence proteins immunity‐related GTPases (IRGs), favours intracellular parasite development (Fentress et al., 2010). TgROP16 phosphorylates signal transducer and activator of transcription STAT3 and STAT6 host factors, in the cell nucleus, leading to host cell immune response downregulation (Ong, Reese, & Boothroyd, 2010; Yamamoto & Takeda, 2012). TgROP38 is responsible for the downregulation of host genes involved in the MAPK signalling pathway and the modulation of host cell apoptosis (Peixoto et al., 2010).

Very few data are available regarding E. tenella ROPKs: only two kinases encoded by loci ETH_00005190 and ETH_00027700, respectively, have been readily identified so far at the proteomic level in sporozoite stage (Oakes et al., 2013); three other ROPKs (encoded by loci ETH_00028855, ETH_00020620, and ETH_00000075) are expressed only in merozoites. The phylogenetic analysis of ROPK sequences from *T. gondii* and E. tenella allowed the identification of four distinct subclades among them the N‐terminal extension (NTE)‐bearing clade containing *Toxoplasma's* ROPKs with homology to the TgROP2 NTE. This clade also comprises the E. tenella ROP kinase encoded by the locus ETH_00005190. The high structural conservation of NTE of the kinase domain suggests a functional selective constraint in NTE‐bearing ROPK (Talevich & Kannan, 2013). Here, we report the first characterisation of an active E. tenella kinase encoded by the locus ETH_00005190 and the importance of the NTE in the catalytic mechanism. We also identified the host p53 protein as a substrate for this parasite kinase.

## 2 RESULTS

### 2.1 Sequence analysis of the ETH_00005190 locus

**Figure 1:**
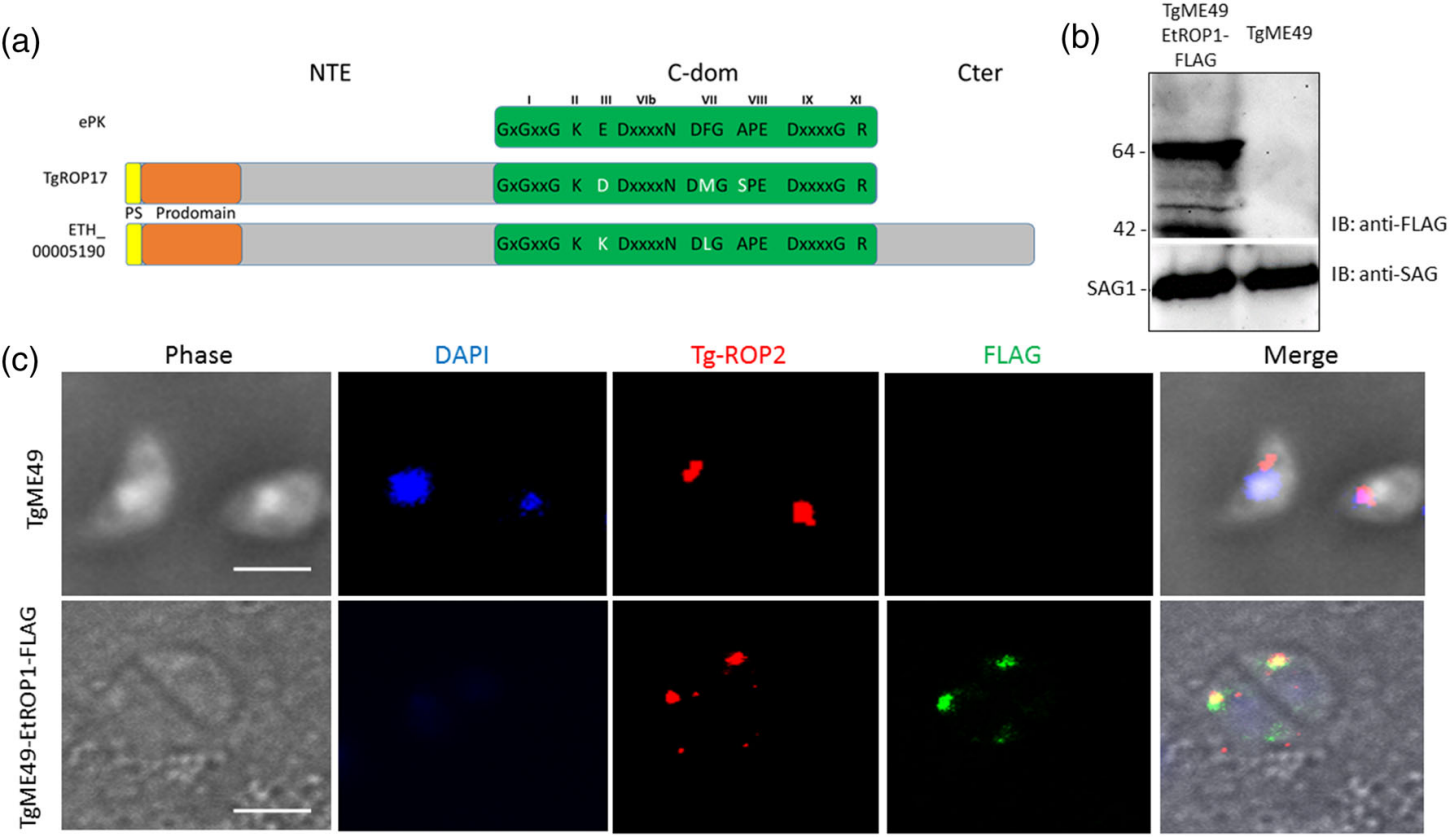
EtROP1 is targeted to the rhoptries and colocalises with TgROP2 in Toxoplasma gondii. (a) ETH_00005190 predicted domains compared to eukaryotic protein kinase (ePK) and ROP17 from T. gondii (TGME49_258580). Conserved subdomains are indicated by Roman numerals above the catalytic domain (C‐dom, green box). The positions of highly conserved amino acids and motifs throughout the ePK superfamily are indicated. Polymorphism between TgROP17 and ETH_00005190 gene products is indicated by white amino acids. Both parasite kinases diverged from ePK by their N‐terminal extension (NTE) and their C‐terminal kinase domain. Adapted from Hanks (2003). PS, peptide signal. (b) Western blot analysis of crude extract from TgME49 and TgME49‐EtROP1‐FLAG tachyzoites. EtROP1‐FLAG expression was only detected in transfected parasites by an anti‐FLAG antibody, which recognises two bands at 64 and 42 kDa. An anti‐SAG1 antibody was used as an internal standard of immunoblot (IB). (c) Fluorescence microscopy analysis of TgME49 and TGME49‐EtROP1‐FLAG tachyzoites showing EtROP1‐FLAG (anti‐FLAG in green) and T. gondii ROP2 localisations (anti‐Tg‐ROP2 in red). Nuclei were stained with nuclear counterstain DAPI (in blue). Bar represents 5 μm

The ToxoDB database was queried in order to gain more insight into the E. tenella locus of interest. The locus ETH_00005190 is annotated as a rhoptry protein kinase (Figures 1a and S1). The predicted protein primary sequence encoded by this locus, sharing 27% identity with TgROP17, contains 665 amino acids resulting in an apparent molecular weight of 73 kDa. It comprises an NTE also observed in TgROP2, 5, 8, 17, and 18 (Figure 1a). The protein contains a N‐terminal predicted signal peptide with a cleavage site between residues 20 and 21 (*p* = .853) and a SɸXE motif at positions 79–82, which is a subtilisin cleavage site conserved in most TgROP sequences (Figure S1). Prosite database predicted a serine/threonine protein kinase domain at residues 257–549, but important residues involved in ATP positioning or in conformational changes necessary for kinase activity were missing: the glutamate and the phenylalanine in subdomain III and VII, respectively, supporting a noncanonical mechanism or the impairment of kinase activity.

### 2.2 EtROP1 is a rhoptry protein

To determine the cellular localisation of the protein, and in absence of any protein usable as positive control, we generated a *T. gondii* ME49 strain expressing ETH_00005190 gene product fused to a FLAG epitope at the 3′ end of the gene, under the TgROP1 5′UTR control (Figure S2). Western blot analysis of recombinant tachyzoites using anti‐FLAG antibodies detected two main bands of 64 and 42 kDa (Figure 1b) corresponding to the expected matured form and a degraded form of the protein, respectively. Immunofluorescence assays showed that ETH_00005190 gene product is localised at the apical pole of the parasite and colocalised with TgROP2 (Figure 1c). These results demonstrate that ETH_00005190 encodes for a rhoptry protein, and the targeting signals are conserved between *Eimeria* and *Toxoplasma*. Based on these features, ETH_00005190 gene product was then renamed EtROP1. EtROP1 retains most of the key residues needed for catalytic activity (excluding the glutamate in subdomain III and the phenylalanine in subdomain VII). This prompted us to investigate EtROP1 enzymatic activity.

### 2.3 EtROP1 is an active kinase and its NTE domain is essential for its catalytic activity

In order to characterise host cell proteins interacting with EtROP1, we chose to express EtROP1‐FLAG in HEK293T (human embryonic kidney) cells for their propensity for transfection.

**Figure 2:**
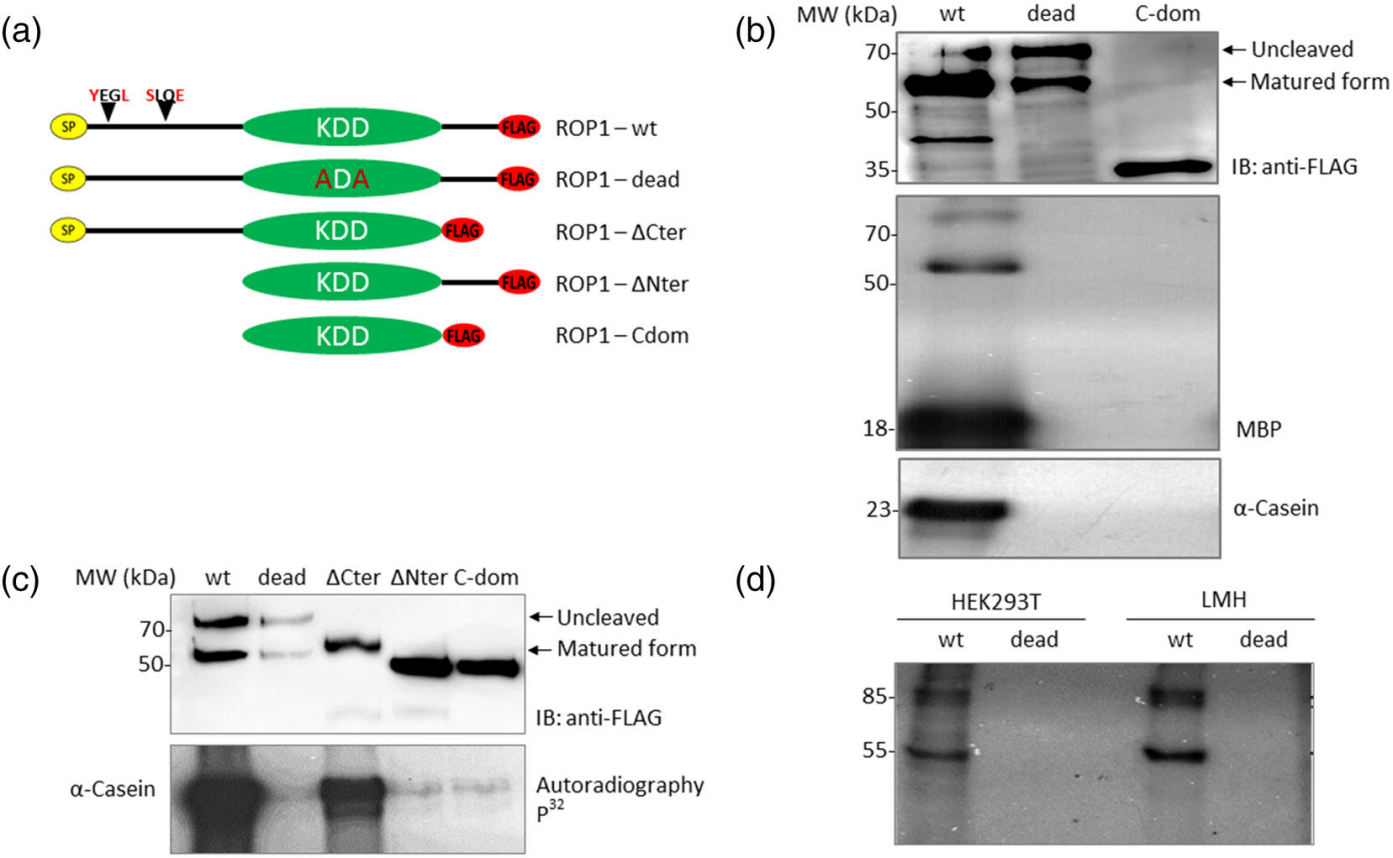
EtROP1 is an active kinase and the NTE is essential for its catalytic activity. (a) Scheme of EtROP1‐FLAG constructs. WT corresponds to the full‐length wild type form of the protein. Dead corresponds to a double mutated construct: the catalytic triad KDD from the catalytic domain was replaced by ADA. ∆Cter corresponds to the protein deleted for its C‐terminal domain (from residue 549 to residue 675) immediately after the catalytic domain. ∆Nter corresponds to the protein deleted for its N‐terminal extension (from residue 1 to residue 257) before the catalytic domain. C‐dom corresponds to the catalytic domain of EtROP1 (from residue 258 to residue 548). All constructs possess a C‐terminal FLAG‐tag for detection. (b) Western blot analysis showing expression of EtROP1 wild type (wt) form, mutant (dead) form and catalytic domain (C‐dom) by HEK293T cells (top panel). SDS‐PAGE autoradiography of a kinase assay performed on EtROP1‐FLAG recombinant kinases (wt, dead and catalytic domain) with MBP (middle panel) and α‐casein substrates (bottom panel). (c) Western blot analysis showing expression of EtROP1 wt, dead, ∆Cter, ∆Nter, and C‐dom forms by HEK293T cells (top panel). SDS‐PAGE autoradiography of a kinase assay performed on EtROP1‐FLAG recombinant kinases (WT, dead, ∆Cter, ∆Nter and C‐dom forms) with the α‐casein substrate (bottom panel). (d) SDS‐PAGE autoradiography of a kinase assay performed on EtROP1‐FLAG recombinant kinases (wt and dead forms) incubated with HEK293T and LMH cell lysates. Two main phosphorylated signals were observed with the EtROP1 wt form, at 55 and 85 kDa

EtROP1 and several truncated forms were expressed in fusion with a C‐terminal FLAG epitope: the wild type (wt), the dead form corresponding to the mutation in the catalytic domain (replacement of KDD from the catalytic triad by ADA), ΔCter and ΔNter forms corresponding to the deletion of the Cter and Nter domain, respectively, and the C‐dom form corresponding to the catalytic domain (Figure 2a). We analysed the kinase activity of the purified EtROP1‐FLAG and truncated forms using [γ‐^32^P]ATP and the heterologous substrates casein and MBP (Figure 2b,c). Results showed that the wild‐type EtROP1 is active and can phosphorylate both casein and MBP substrates in vitro. However, the catalytic domain alone and the dead mutant failed to phosphorylate casein or MBP. Unlike the C‐terminal domain, the NTE domain of EtROP1 was essential for kinase activity (Figure 2c). Autophosphorylation was observed for active forms of EtROP1, and mass spectrometry analysis indicated that the phosphorylation sites were serine residues located at C‐terminal part of the kinase (data not shown). EtROP1 is therefore the first ROPK characterised in *Eimeria*.

In order to identify parasite or host cell EtROP1 partners, purified recombinant EtROP1 was incubated with a host cell lysate (HEK293T and LMH, a primary avian hepatocellular carcinoma epithelial cell line) in a kinase activity assay (Figure 2d). Two major phosphorylated signals at 85 and 55 kDa were observed in cell lysates, regardless of the cell lines. The absence of signal with the dead mutant confirmed that the observed phosphorylation results from EtROP1 activity. To identify EtROP1 host cell substrates, an immunoprecipitation assay was performed.

### 2.4 EtROP1‐FLAG interacts with p53 via its N‐terminus part

We performed a co‐immunoprecipitation to isolate native complexes from transiently transfected HEK293T cells expressing EtROP1‐FLAG. Purification of EtROP1‐FLAG‐containing complexes (Figure 2d) and subsequent LC‐MS/MS analysis identified approximately 29 proteins in the 55‐kDa signal among which, the tumour protein p53 (protein ID gi_112489878; Table S1), modulating apoptosis in response to multiple stress signals (Levine, 1997). Our result, and previous observations reporting apoptosis inhibition in host cells infected by *Eimeria* (del Cacho, Gallego, Lopez‐Bernad, Quilez, & Sanchez‐Acedo, 2004; Lang, Kann, Zahner, Taubert, & Hermosilla, 2009; Major et al., 2011), prompted us to focus on this candidate.

**Figure 3:**
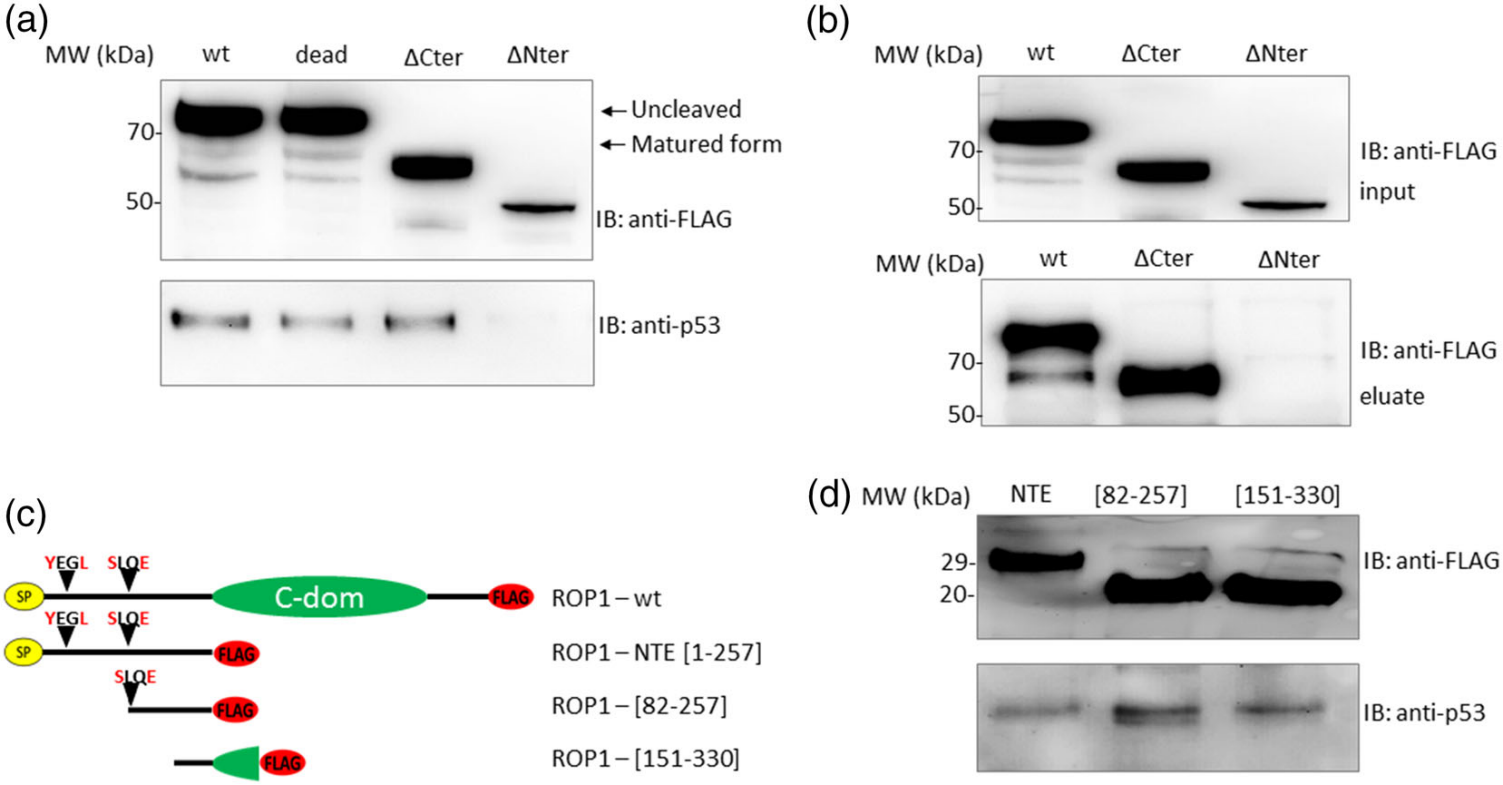
EtROP1 coprecipitates p53 via its NTE domain. (a) Western blot analysis showing host‐cell p53 co‐purification with EtROP1‐FLAG wt, dead, ∆Cter or ∆Nter forms in a pull‐down assay using anti‐FLAG affinity‐chromatography after constructs transfection in HEK293T cells. EtROP1‐FLAG constructs (wt, dead, ∆Cter and ∆Nter; top panel) and p53 (bottom panel) were detected using an anti‐FLAG or an anti‐p53 antibody, respectively. (b) Western blot analysis showing EtROP1‐FLAG co‐purification with a 6His‐tagged p53 recombinant protein in a pull‐down assay. Protein lysates from HEK293T cells transfected with EtROP1‐FLAG constructs (wt, ∆Cter and ∆Nter) (input, top panel) and eluted complexes (eluate, bottom panel) were both detected using an anti‐FLAG antibody. (c) Scheme of EtROP1‐FLAG constructs. NTE corresponds to the N‐terminal extension domain of the protein (residues 1 to 257), [82–257] corresponds to the amino acid sequence between the maturation site (SLQE) and the beginning of the catalytic domain (residues 82 to 257) and [151–330] corresponds to the end of the NTE domain and the beginning of the catalytic domain (residues 151 to 330). (d) Western blot analysis showing host‐cell p53 co‐purification with EtROP1‐FLAG NTE, [82–257] and [151–330] forms in a pull‐down assay using anti‐FLAG affinity‐chromatography after constructs transfection in HEK293T cells. EtROP1‐FLAG constructs (NTE, [82–257] and [151–330]; top panel) and p53 (bottom panel) were detected using an anti‐FLAG or an anti‐p53 antibody, respectively

EtROP1‐p53 interaction was further confirmed by a pull down experiment (Figure 3). Co‐purification of p53 and EtROP1‐FLAG was observed with EtROP1 wt, dead mutant and ΔCter constructs, but not with the ΔNter form (Figure 3a), suggesting that the Nter domain is essential for EtROP1‐p53 interaction. This result was further confirmed by a reverse pull down assay in which 6His‐p53 immunoprecipitated EtROP1 wt and ΔCter constructs but not the ΔNter form (Figure 3b). In order to precisely define the interaction domain within the NTE, we created three new constructs corresponding to (a) the entire NTE, (b) the amino acid sequence between the maturation site (SLQE) and the catalytic domain (residues 82–257), and (c) the end of the NTE domain and the beginning of the catalytic domain (residues 151–330) corresponding to a highly conserved region among *Eimeria* species (Figure 3c). The p53 protein was immunoprecipitated with all constructs (Figure 3d), suggesting that the minimal interaction domain is comprised between residues 151–257. Taken together, these results clearly indicate that the Nter domain of EtROP1 is essential for interaction with p53.

### 2.5 Host cell p53 is phosphorylated by EtROP1

**Figure 4:**
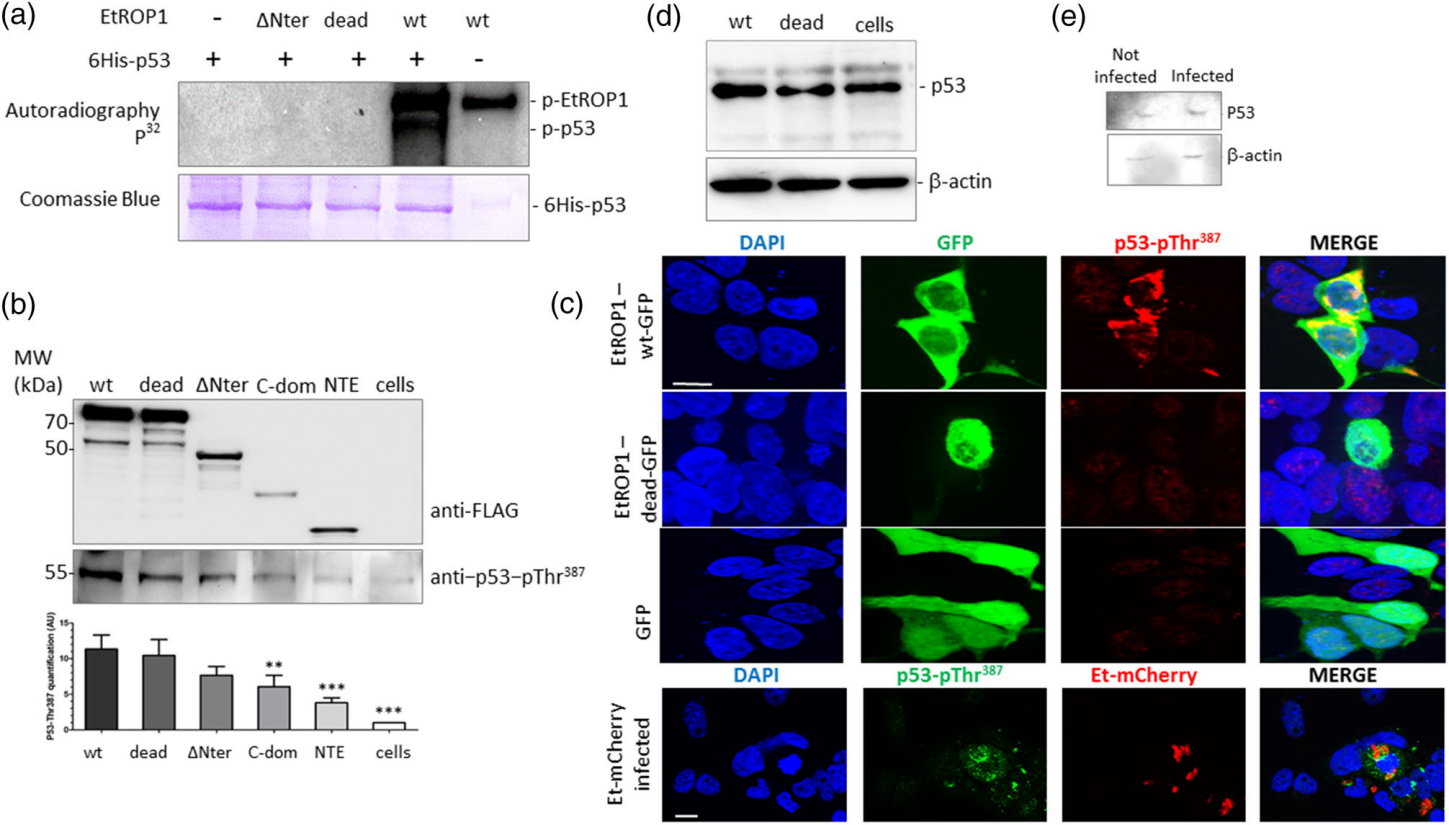
EtROP1 phosphorylates p53 on residue Thr^387^. (a) SDS‐PAGE autoradiography of a kinase assay (top panel) performed on EtROP1‐FLAG recombinant proteins (WT, dead and ∆Nter forms) and a 6His‐tagged p53 recombinant protein (detected by SDS‐PAGE followed by Coomassie blue staining, bottom panel). (b) Western blot analysis showing p53 phosphorylated Thr^387^ residue (p53‐pThr^387^) co‐purification with EtROP1‐FLAG wt, dead, ∆Nter, C‐dom and NTE forms in a pull‐down assay using anti‐FLAG affinity‐chromatography after constructs transfection in HEK293T cells. EtROP1‐FLAG constructs (wt, dead, ∆Nter, C‐dom and NTE; top panel) and p53‐pThr^387^ (bottom panel) were detected using an anti‐FLAG or an anti‐p53‐pThr^387^ antibody, respectively. Western blot signal were quantified by densitometry and ImageJ. (c) Upper panel: fluorescence microscopy analysis showing GFP (green) and phosphorylated p53 (anti‐p53‐pThr^387^ antibody, red) detection in HEK293T cells transfected with EtROP1‐GFP expression plasmids (wt and dead forms) or the control plasmid pcDNA‐GFP. Lower panel: phosphorylated p53 (anti‐p53‐pThr387 antibody, green) detected in HEK293T cells infected with E. tenella expressing mcherry (red). Bar represents 10 μm. (d) Western blot analysis showing p53 stabilisation in HEK293T cells transfected with EtROP1‐GFP expression plasmids (wt and dead forms) or the control plasmid pcDNA‐GFP. Two days posttransfection, GFP positive cells (transfected cells) were flow cytometry sorted before immunoblot analysis using an anti‐p53 antibody. An anti‐β‐actin antibody was used as an internal standard of immunoblot (IB). (e) Western blot analysis showing p53 stabilisation in epithelial caecal cells infected with E. tenella. Eighty‐four hours postinfection, mCherry positive caecal cells (infected cells) were flow cytometry sorted for subsequent total RNA purification. Analysis was run as in 4D legend

The protein p53 is regulated by several mechanisms, among which phosphorylation. After demonstrating that p53 interacts with EtROP1, we performed a kinase assay and were able to demonstrate that EtROP1 phosphorylates a recombinant p53 (Figure 4a). As expected, EtROP1 dead and ΔNter forms had no kinase activity towards p53.

In order to identify p53 residues phosphorylated by EtROP1, a kinase assay was achieved and subsequent phosphopeptides were further analysed. Thr^387^ was the only phosphorylated residue identified by mass spectrometry (Table S2). To confirm this result, a western blot analysis was performed on HEK293T lysates following transient transfection with EtROP1 constructs (Figure 4b). Even though a background signal was observed in all samples, probably due to endogenous cellular kinases, quantification of signal indicated that phosphorylation of Thr^387^ was significantly increased in HEK293T expressing the wt EtROP1.

An immunostaining of the phosphorylated Thr^387^ was also performed on transiently transfected HEK293T with wt EtROP1. A clear staining around host cell nucleus was observed with the wt EtROP1 but not with the dead form of the protein (Figure 4c). This result was also confirmed in HEK293T cells infected by recombinant E. tenella strain expressing mCherry fluorescent marker. Anti‐P53‐pThr^387^ generates a punctuated signal in the cytoplasm of infected cells, whereas no staining was detected in uninfected GFP control cells (Figure 4c, lower panel). The phosphorylation of Thr^387^ is associated with p53 stabilisation in cells overexpressing wt EtROP1 (Figure 4d) and also in cells infected by E. tenella (Figure 4e). Western blot signal quantification and standardisation with β‐actin signal indicated that p53 abundance was increased by 44% in infected cells (Figure 4e). Altogether, these results strongly support p53 phosphorylation by EtROP1, on residue Thr^387^.

### 2.6 EtROP1 inhibits host cell apoptosis

E. tenella infection results in inhibition of host cell apoptosis (Lang et al., 2009). Because EtROP1 can interact with and phosphorylate the apoptosis regulator p53, we hypothesised that EtROP1 may be involved in the modulation of host cell apoptosis. To investigate this hypothesis, we analysed the level of apoptosis in HEK293T cells transiently transfected with EtROP1 constructs. Cell apoptosis was induced by actinomycin D treatment (blocking RNA synthesis). Apoptosis was then evaluated at 24‐ or 48‐hr postinduction using annexin V‐PE staining and caspase activity assay.

**Figure 5:**
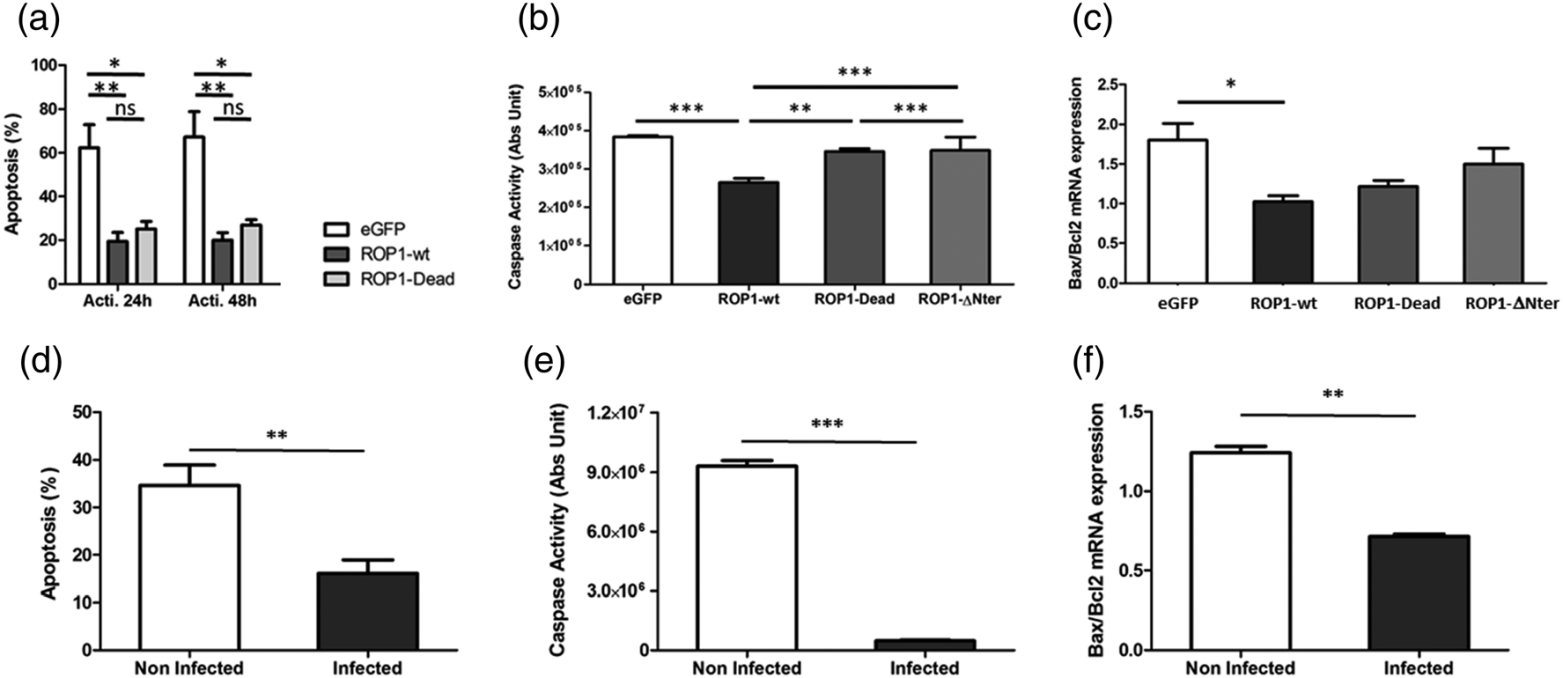
EtROP1 inhibits host cell apoptosis. (a) Annexin V‐PE quantification by FACS analysis of HEK293T cells transfected with EtROP1‐GFP expression plasmids (wt and dead forms) or the control plasmid pcDNA‐GFP. Two days posttransfection, cell apoptosis was induced by actinomycin D. After 24 hr or 48 hr postapoptosis induction, Annexin V‐PE and efluor780 staining were performed and GFP positive cells (transfected cells) were flow cytometry sorted. Apoptotic cells (Annexin V‐PE stained) were quantified. Necrotic cells were stained by efluor780 and excluded from the analysis. Global ANOVA analysis was significant for EtROP1 constructs (p < .0001). Different means between pairs of sample groups were analysed by a two‐way ANOVA. (b) Caspase 3/7 activity in HEK293T cells transfected with EtROP1‐GFP expression plasmids (wt and dead forms) or the control plasmid pcDNA‐GFP. Two days posttransfection, GFP positive cells (transfected cells) were flow cytometry sorted and the caspase activity measured using the fluorogenic z‐DEVD caspase 3/7 substrate and a Glomax photometer. ANOVA analysis was significant (p < .0001). Different means between pairs of sample groups were analysed by a Newman–Keuls Multiple Comparison Test. (c) Bax/Bcl_2_ gene expression quantified by RT‐qPCR in HEK293T cells transfected with EtROP1‐GFP expression plasmids (wt and dead forms) or the control plasmid pcDNA‐GFP. Two days posttransfection, GFP positive cells (transfected cells) were flow cytometry sorted for subsequent total RNA purification. Gene expression values were normalised to the human housekeeping β‐actin and GAPDH transcripts. Values are expressed as fold increase versus non transfected cells. Different means between pairs of sample groups were analysed by a one‐way ANOVA. (d) Annexin V‐PE quantification by FACS analysis of epithelial cells from caeca infected with mCherry E. tenella recombinant strain. Eighty‐four hours postinfection, Annexin V‐PE and efluor780 staining were performed and mCherry positive cells (infected cells) were flow cytometry sorted. Analysis was run as mentioned in 5A legend. (e) Caspase 3/7 activity in epithelial cells from caeca infected with mCherry E. tenella recombinant strain. Analysis was run as mentioned in 5B legend. (f) Bax/Bcl_2_ gene expression quantified by RT‐qPCR in epithelial cells from caeca infected with mCherry E. tenella recombinant strain. Gene expression values were normalised to the avian housekeeping β‐actin, G10, and GAPDH transcripts. Values are expressed as fold increase versus non infected epithelial cells. Different means between samples were analysed by a one‐way ANOVA

During apoptosis, phosphatidylserines localised in the inner side of cell membranes are shifted to the cell surface, leading to a loss of membrane asymmetry. Annexin V‐PE staining of phosphatidylserines was reduced in HEK293T cells expressing EtROP1. EtROP1 expression significantly reduced host cell apoptosis, but this result was independent from its kinase activity as both wt and dead forms reduced host cell apoptosis (Figure 5a). We also used caspase activation as an indicator of apoptosis. Caspases 3/7 activities were reduced by both active and dead EtROP1 forms (Figure 5b). The pro‐apoptotic protein Bax and the antiapoptotic protein Bcl_2_ are transcriptional targets for p53. The ratio Bax/Bcl_2_ is a determinant of cell survival or apoptosis. We decided to investigate Bax and Bcl_2_ gene expression. HEK293T cells showed significantly lower Bax/Bcl_2_ ratio (Figure 5c) when transfected with the wt EtROP1. The dead EtROP1 gave the same result as the wt EtROP1 suggesting another functional action of EtROP1 on p53 in phosphorylation independent manner. Taken together, these results indicate that the EtROP1‐mediated apoptosis inhibition is independent from its catalytic activity. Our findings were confirmed in avian cell lines (Figure S3). In chicken lung epithelial cell lines (CLEC‐213), overexpression of EtROP1 was associated with reduction in caspases 3/7 activities and tends to reduce the ratio Bax/Bcl_2_ expression. In order to confirm those findings during E. tenella infection in vivo, apoptosis was analysed in infected epithelial cell from the caecum from infected chicken. E. tenella infection significantly inhibits host cell apoptosis (Figure 5d–f). Altogether, these results strongly support that EtROP1 inhibits host cell apoptosis during intracellular development of *E. tenella*.

### 2.7 EtROP1 induces host cell arrest in G0/G1 through the activation of p53/p21 pathway

**Figure 6:**
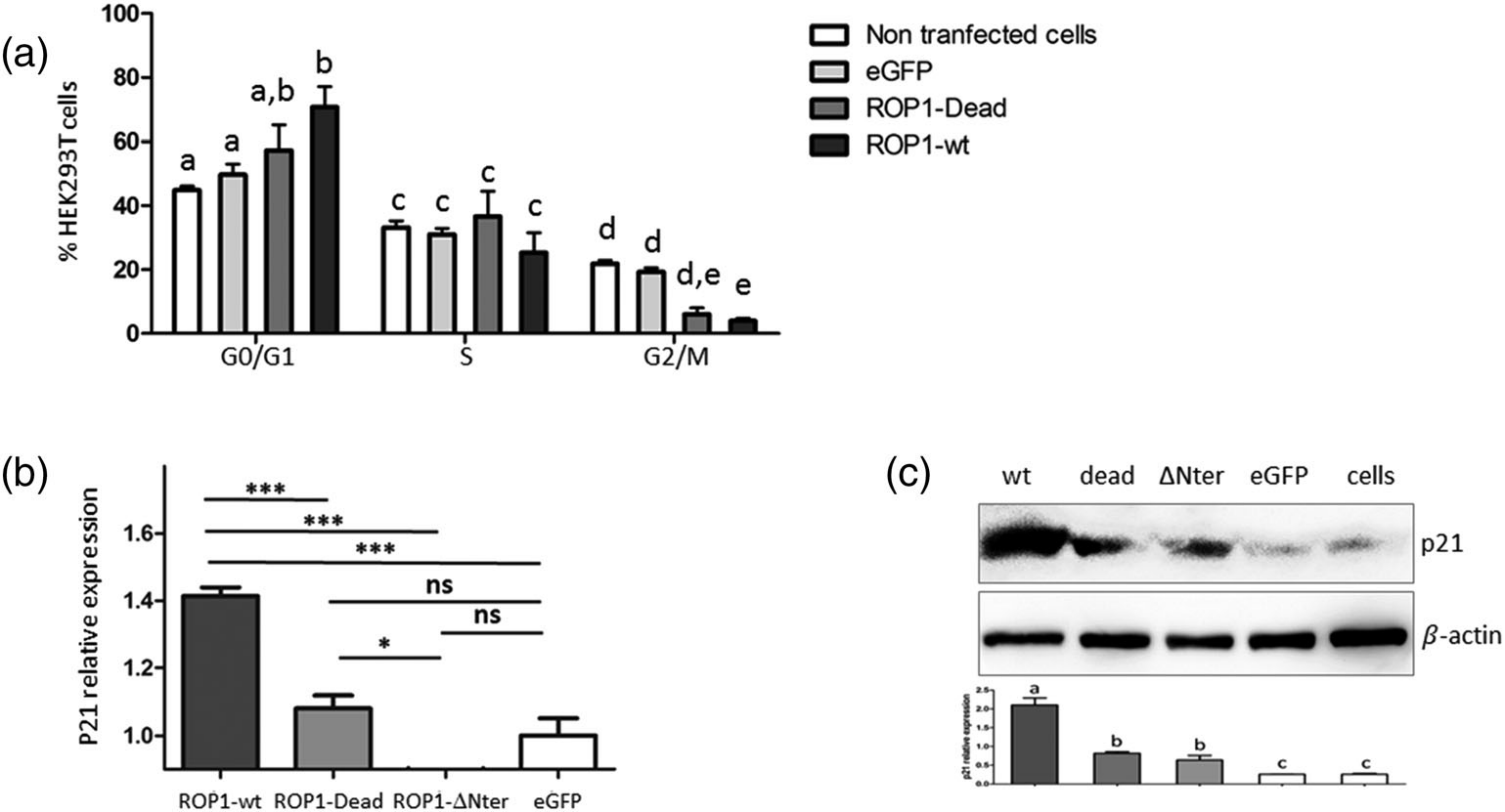
EtROP1 induces G0/G1 cell cycle arrest in HEK293T cells. (a) Cell cycle distribution of HEK293T cells nontransfected (NT cells), transfected with the control plasmid pcDNA‐GFP (GFP) or with EtROP1‐GFP expression plasmids (wt and dead forms). Two days posttransfection, GFP positive cells (transfected cells) were flow cytometry sorted using propidium iodide staining to assess the percentage of cells in each phase (G0/G1, S, G2/M). Data represent the average from three independent experiments. Differences in cell cycle phases between sample groups were analysed by a chi‐squared test. Different letters refer to different statistical groups. (b) p21 gene expression determined by RT‐qPCR in HEK293T cells transfected with EtROP1‐GFP expression plasmids (wt and dead forms) or the control plasmid pcDNA‐GFP. Two days posttransfection, GFP positive cells (transfected cells) were flow cytometry sorted for subsequent total RNA purification. Gene expression values were normalised to the human housekeeping β‐actin and GAPDH transcripts. Values are expressed as fold increase versus pcDNA‐GFP transfected cells. Different means between pairs of sample groups were analysed by a one‐way ANOVA. (c) Western blot analysis showing p21 detection in HEK293T cells transfected with EtROP1‐GFP expression plasmids (wt and dead forms) or the control plasmid pcDNA‐GFP. Two days posttransfection, GFP positive cells (transfected cells) were flow cytometry sorted before immunoblot analysis to detect p21. An anti‐β‐actin antibody was used as an internal standard. Western blot signal were quantified by densitometry and ImageJ. Different letters refer to different statistical groups

To evaluate the effects of EtROP1 expression on host cell cycle, we transfected HEK293T with a GFP‐tagged EtROP1. Flow cytometry analysis of transfected cells demonstrated that HEK293T expressing EtROP1‐GFP were arrested in G0/G1 phase (Figure 6a). Cells arrested in G0/G1 phase were significantly more abundant when transfected with the wt EtROP1 in comparison with nontransfected cells, cells transfected with an empty plasmid, or with the dead kinase (*p* = .001, *p* = .002, and *p* = .008, respectively). The dead EtROP1 had no effect on host cell arrest compared with cells transfected with empty plasmid. Host cell cycle is governed by cyclin‐dependent kinases, which are themselves regulated by the kinase inhibitor protein family including p21. We decided to investigate p21 gene expression during EtROP1‐mediated cell cycle modulation. HEK293T cells showed significantly higher p21 (Figure 6b) gene expression when transfected with the wt EtROP1. In addition, neither the dead nor the ΔNter forms modified p21 transcription level. Then, we assessed p21 expression at the protein level (Figure 6c). Western blot analysis of transfected HEK293T cells confirmed the significant increase of p21 expression at the protein level for the wt EtROP1 (Figure 6c). This EtROP1‐mediated p21 overexpression is consistent with the previously observed host cell cycle arrest. Taken together, our results suggest that EtROP1 expression induces cell cycle arrest through the activation of the p53/p21 pathway. To confirm those findings on epithelial chicken cells, we transfected avian cells with a GFP‐tagged EtROP1. As for human cells, flow cytometry sorted avian cells showed a significant increase of cells arrested in G0/G1 phase when transfected with wt EtROP1 (Figure S4). The dead EtROP1 had no effect on host cell arrest. Avian cells showed significantly higher p21 gene expression when transfected with the wt EtROP1 and the mutated EtROP1, conversely to truncated forms that had no effects on P21 expression. Those findings were confirmed in E. tenella infected epithelial cell from the caecum (Figure S4).

## 3 DISCUSSION

We describe the identification and functional characterisation of the first ROP kinase from *Eimeria tenella*, a putative virulence factor. In an overexpression model using HEK293T, EtROP1 interacts with and phosphorylates host cell p53 on Thr^387^, leading to p53 stabilisation. Using actinomycin D, which blocks mRNA synthesis and induces apoptosis, we showed that EtROP1 (wt and dead) prevents apoptosis in a manner that is independent of its kinase activity. This is an additional evidence that ROPK plays some biological functions in kinase activity independent manner. The pseudokinase TgROP5, which is catalytically null, plays an important role in *T. gondii* virulence (Behnke et al., 2012). TgROP5 accumulates in PVM and avoids the IRG‐GTPase accumulation (Niedelman et al., 2012; Reese & Boyle, 2012). Our data were also confirmed with doxorubicin, blocking protein synthesis (data not shown). The results obtained with the heterologous system of HEK293T cells were confirmed in avian cell lines with transfection and with E. tenella infection. ROP kinases are characterised by their sequence diversity in comparison with eukaryote protein kinases superfamily. Amino acids or motifs usually conserved in the catalytic domain of protein kinases (Hanks, 2003) may be missing for both pseudokinases and active ROP kinases. These particular characteristics suggest a noncanonical catalytic mechanism. An NTE domain is present in several active and inactive ROPK from *T. gondii*, and a small number of residues show a strong conservation (Talevich & Kannan, 2013). Pseudokinase TgROP5 increases the in vitro phosphorylation of IRGs by the active TgROP18 and is required for effective phosphorylation of Irgb6 by TgROP18 in the context of infection, suggesting an important function for TgROP5‐NTE because TgROP5 is catalytically inactive (Behnke et al., 2012; Fleckenstein et al., 2012). Mechanistic studies have shown the importance of this NTE domain in the interaction between *T. gondii* ROP and their host cell substrates: TgROP18 and ATF6 (Yamamoto & Takeda, 2012) or TgROP16 and STAT3 (Yamamoto et al., 2009). Although some residues are conserved within NTE sequences from *T. gondii* ROPKs, no residue conservation could be detected within NTE domain in comparison with EtROP1, which shows that variability of the NTE sequence is consistent with specificity of substrate binding, and finally with the specificity of host cell interaction. In the present study, we revealed that EtROP1 NTE is involved in the interaction with the host cell p53 and is required for the enzyme catalytic activity.

Host cell p53 was identified as one of the EtROP1 interacting proteins. Interestingly, this major intracellular regulator is involved in cell cycle regulation and stress‐induced apoptosis. EtROP1 overexpression was associated with p53 stabilisation and phosphorylation of residue Thr^387^ (located in the negative regulation domain, at the C‐terminal region of p53). Transfection leads to overexpression of EtROP1, which might exacerbates the phenotypes observed. However, at a lower intensity, the phosphorylation of p53 and its stabilisation were confirmed in E. tenella infected cells. Thr^387^ is a physiological target of the cell cycle checkpoint kinase CHK1 (Ou, Chung, Sun, & Shieh, 2005), which could explain the observed G0/G1 cell cycle arrest (Kastan & Bartek, 2004) in HEK293T cells. It is well known that p53 can promote cell cycle arrest through p21 activation (El‐Deiry et al., 1993), leading to G0/G1 arrest, preventing S phase entry. This is fully consistent with our findings: EtROP1 wt overexpression induced G0/G1 arrest as well as a reduction in the S phase cell population and an induction of p21 expression, hallmarks of p53 activation. However, overexpression of a catalytically inactive EtROP1 had no effect on the cell cycle, which means that the kinase activity is required for cell cycle arrest but not for apoptosis inhibition.

Regulation of p53 function is tightly controlled through several mechanisms, including transcription, translation, posttranslational modification, and subcellular localisation. Our data support that EtROP1 may be responsible for the antiapoptotic activity of E. tenella in host cells through p53 stabilisation. Different explanations are possible: p53 is sequestrated in the cytoplasm (Haller et al., 2010) or EtROP1 is part of a parasite protein complex in which another active kinase is responsible for p53 phosphorylation. The immunostaining of p53‐pThr^387^ in the cytoplasm of infected cells supports the former hypothesis.

Apicomplexan parasites interfere with the apoptotic process of infected cells: after an early inhibition, parasites can induce host cell apoptosis. E. tenella may have this dual activity. In the early phase of infection, previous in vivo studies (del Cacho et al., 2004; Lang et al., 2009; Lee, Hong, Chung, & Kim, 2012) and present data demonstrate that inhibition of host cell apoptosis by E. tenella is likely to favour E. tenella development, ensuring survival within the host cell and allowing maturation of schizonts. In the second phase of infection, the mature schizont induces host cell apoptosis favouring merozoites release (del Cacho et al., 2004) by means of a calcium‐dependent mechanism (Cui et al., 2016). This dual activity was also described for other apicomplexan parasites such as *Plasmodium*, *Toxoplasma*, *Theileria*, *Cryptosporidium* (Goebel, Gross, & Luder, 2001; Guergnon, Dessauge, Langsley, & Garcia, 2003; Heussler et al., 1999; Leiriao et al., 2005; McCole, Eckmann, Laurent, & Kagnoff, 2000; Nash et al., 1998; ToureBalde et al., 1996) supporting a mechanism shared within the phylum (Luder, Stanway, Chaussepied, Langsley, & Heussler, 2009). The mechanism by which parasite protects cells from induction of apoptosis has been partially deciphered: in *T. gondii* model, the dense granule TgGRA16 alters host p53 levels, in a HAUSP‐dependent manner (Bougdour et al., 2013; Chang et al., 2015). However, dense granules were not observed by electronic microscopy in *Eimeria* parasites, and genes encoding GRA proteins were not detected within their genomes, leading to the hypothesis that TgGRA16 and TgROP16 role in host cell apoptosis regulation observed in *T. gondii* may be played by EtROP1 in E. tenella. Apoptosis is also induced during *T. gondii* infection. TgGRA15, TgROP16, and TgROP18 were involved in triggering neuroblastomal and choriocarcinoma apoptosis (An et al., 2018; Chang et al., 2015; Wan et al., 2015; Wei et al., 2018). Induction of apoptosis in immune cells (lymphocytes, natural killers, and macrophages) contributes to evasion of the host's immune defence during the acute phase of infection. In the central nervous system, the most commonly affected organ, parasites may trigger apoptosis of long‐lived cells (i.e., half‐life of 7 years; Spalding et al., 2013) to induce egress. This is very different from E. tenella that develops in digestive epithelial cells characterised by a 4–5 days turnover. This may explain the different role of ROPK in a neurotropic parasite versus a digestive epithelial parasite.

EtROP1 is highly conserved between avian *Eimeria* species (43% to 92% identity, data not shown), and we may speculate that EtROP1 has crucial functions, probably shared by species infecting avian hosts. Interestingly, inhibition of host cell apoptosis by sporozoites has been described for E. tenella (del Cacho et al., 2004) but also for Eimeria bovis (Lang et al., 2009) and *Eimeria acervulina* (Major et al., 2011), which present, compared with the caecal species E. tenella, distinct sites of infection (ileum and duodenum, respectively) and distinct developmental cycles (21 and 5 days, respectively).

Elucidation of the mechanisms involved in host cell apoptosis inhibition by *Eimeria* harbours the potential of identifying new parasite factors hijacking host cell apoptosis pathway, which may represent new targets for devising control strategies against coccidiosis. Moreover, EtROP1 deletion in *Eimeria* by transgenesis would be relevant to confirm its function in vivo. Unfortunately, genetic manipulation in *Eimeria* has been limited to knock‐in strain production so far (Clark et al., 2008; Clark et al., 2012; Marugan‐Hernandez et al., 2016; Rieux et al., 2012). The CRISPR‐Cas9‐mediated genome editing approach should soon keep its promises and allow the generation of knockout virulence‐attenuated strains that may be valuable for vaccination.

## 4 EXPERIMENTAL PROCEDURES

### 4.1 Parasites and cell culture

*T. gondii* tachyzoites of the ME49 strain (Biological Resource Center BRC Toxoplasma, Limoges, France) were maintained by serial passages in human foreskin fibroblasts (HFF) monolayers (ATCC® SCRC1041).

*E. tenella* Wisconsin strain (Wis) expressing yellow fluorescent protein (YFP) or *E. tenella* INRA strain expressing mCherry fluorescent protein (Bussiere et al., 2018; Gras et al., 2014) were maintained by serial passages in chicken. Freshly excysted sporozoites were purified as described (Bussiere et al., 2015).

Human embryonic kidney cells (HEK293T, ATCC® CRL3216) and HFF were cultured at 37°C under 5% CO_2_ in Dulbecco's Modified Eagle's medium (DMEM) containing 10% fetal calf serum (Gibco‐Invitrogen, Carlsbad), 2‐mM L‐glutamine, 50‐U/ml penicillin, and 50‐μg/ml streptomycin. Avian epithelial cell lines (liver epithelial cells LMH, ATCC® CRL2117 and lung epithelial cell CLEC‐213; Esnault et al., 2011) were cultured at 41°C under 5% CO_2_ in William's medium and Dulbecco's Modified Eagle's medium (DMEM/F‐12), respectively, containing 10% fetal calf serum (Gibco‐Invitrogen, Carlsbad), 50‐U/ml penicillin, and 50‐μg/ml streptomycin.

### 4.2 In vivo E. tenella infection

Outbred PA12 chickens aged 4 to 6 weeks were infected orally with 7.5 × 10^5^ sporulated oocysts of the recombinant E. tenella mCherry strain. Chickens were euthanised at 84 hr p.i., and caeca were recovered. Caecal tissues were incubated with collagenase H (1 mg/ml) at 250 rpm, at 37°C for 30 min. Infected and noninfected epithelial cell from the caecum were stained for cell cycle analysis. Then mCherry‐positive cells (i.e., infected cells) and mCherry‐negative cells (i.e., noninfected cells) were separately sorted by flow cytometry on a MoFlo Astrios EQ (Beckman Coulter) high‐speed cell sorter.

### 4.3 Oligonucleotides

*E. tenella*‐specific primers were designed on the basis of nucleotide sequences collected from ToxoDB (http://toxodb.org). Human‐ and avian‐specific primers were designed on the basis of nucleotide sequences collected from NCBI (https://www.ncbi.nlm.nih.gov/). All primers were purchased from Eurogentec.

### 4.4 Sequence analyses

Homology analyses were performed using BLAST (http://www.ncbi.nlm.nih.gov/blast/) and the Conserved Domain Database CD‐Search (http://www.ncbi.nlm.nih.gov/Structure/cdd/wrpsb.cgi) with default settings. Proteins were aligned using MultAlin (Corpet, 1988). Potential signal peptide cleavage sites were identified with SignalP 3.0 (http://www.cbs.dtu.dk/services/SignalP/; Bendtsen, Nielsen, von Heijne, & Brunak, 2004). Potential alpha helices were identified using PSIPRED v3.0 (http://bioinf.cs.ucl.ac.uk/psipred/; Buchan, Minneci, Nugent, Bryson, & Jones, 2013) and PSSpred (http://zhanglab.ccmb.med.umich.edu/PSSpred/; Yan, Xu, Yang, Walker, & Zhang, 2013). Functional domains were predicted using Prosite (Sigrist et al., 2010).

### 4.5 Plasmid construction

The following EtROP1 (ETH_00005190) constructs were produced: the wild‐type form (WT), the inactive form (Dead) in which the catalytic triad KDD was replaced by ADA, the truncated forms ΔNter and ΔCter, the catalytic domain alone (C‐dom), the NTE alone, and two truncated forms of the NTE ([82–257] and [151–330] regions). The inactive form (Dead) mutated in K285A and D404A was generated with a QuikChange Site‐Directed Mutagenesis kit (Stratagene). To construct EtROP1 expression vectors, all EtROP1 (full length or truncated) sequences were amplified by PCR using the polymerase Phusion‐High Fidelity (Fermentas) and cloned between *BamHI* and *NotI* sites on the pcDNA3 (Invitrogen) expression vector in frame with a C‐term FLAG‐tag or between *BamHI* and *XhoI* sites on the pcDNA3 expression vector in fusion with a C‐term GFP‐FLAG‐tag. All primers used are listed in Table S3.

### 4.6 Parasite transfection and selection

We generated a *T. gondii* ME49 strain expressing ETH_00005190 flanked with a C‐term FLAG epitope and under TgROP1 5′UTR control. TgROP1 5′UTR sequence was amplified from *T. gondii* ME49 genomic DNA. EtROP1 (ETH_00005190) was amplified from E. tenella (Wisconsin strain) genomic DNA and subcloned into the p230‐2G vector (generous gift from VitamFero). Transgenic *T. gondii* expressing EtROP1‐FLAG under TgROP1 promoter were obtained by electroporation of pT230‐2G‐EtROP1 vector into *T. gondii* ME49 tachyzoites and grown on HFF. After 24 hr, transfectants were selected by addition of phleomycin (5 μg/ml) for 6 hr and 3 days later. The recombinant strain was called TgME49‐EtROP1‐FLAG (Figure S2).

### 4.7 Cell transient transfection experiment

Transfection was carried out using FuGENE6® (Promega) according to manufacturer's instructions. Briefly, T75 flasks of cells grown at 90% confluence were transfected with 10 μg of pcDNA vectors and grown for 2–3 days. Cells were then used for purification of FLAG‐tagged recombinant proteins, flow cytometry analysis (see below), or qRT‐PCR analysis.

### 4.8 Co‐immunoprecipitation

Transfected cells were lysed in lysis buffer containing 20‐mM Tris–HCl (pH 7.5), 1‐mM EDTA, 150‐mM NaCl, 0.1% Nonidet P‐40, and EDTA‐free complete protease inhibitor cocktail (Roche). Lysates were centrifuged and incubated with agarose‐conjugated rabbit polyclonal M2 (anti‐FLAG) antibodies (Sigma). Beads were then washed three times with lysis buffer, and immunocomplexes were eluted in 100 μl with M2 peptide. The binding protein complexes were detected on Coomassie‐stained SDS‐PAGE gels. The gel lane of interest was cut into slices, in‐gel tryptic digested and processed for mass spectrometry analysis (see below). For p53 analysis, the binding protein complexes were analysed on SDS‐PAGE gels and immunoblots using an anti‐p53 antibody (sc99, Santa Cruz).

### 4.9 Mass spectrometry analysis

Proteins were subjected to proteolysis using trypsin, and peptides were analysed by an LTQ Orbitrap Velos mass spectrometer (Thermo Electron) coupled with a nano‐UHPLC Ultimate 3000 RSLC (Thermo Electron). Data were processed using Mascot Distiller v2.2 and Mascot Daemon 2.3 (Matrix Science). Mascot results obtained from the target database searches, with human and the alveolata and the NCBI nonredundant database (December 2015), were subjected to Scaffold software (Proteome Software Inc., Portland, USA). Peptide identifications were accepted, if they could be established at greater than 95% probability by the Peptide Prophet algorithm (Keller, Nesvizhskii, Kolker, & Aebersold, 2002). Protein identifications were accepted if they could be established at greater than 95% probability and contained at least two unique peptides. Protein probabilities were assigned by the Protein Prophet algorithm (Nesvizhskii, Keller, Kolker, & Aebersold, 2003). Proteins that contained similar peptides and could not be differentiated on the basis of MS/MS analysis alone were grouped to satisfy the principles of parsimony. Proteins sharing significant peptide evidence were grouped into clusters. Functional annotations and quantifications of identified proteins were explored using ScaffoldMS R package (https://forgemia.inra.fr/aurelien.brionne/ScaffoldMS).

### 4.10 Expression of a 6‐histidine‐tagged p53 in E. coli

The coding sequence of human p53 was cloned into the pET15b expression vector, in frame with a 6His tag between *NdeI* and *BamHI* sites using the following primers: forward primer 5′GGGCCCCATATGATGGAGGAGCCGCAGTCAGATC3′ and reverse primer 5′GACAGAAGGGCCTGACTCAGACTGAGGATCCGGACCT3′. The resulting construct was used to transform E. coli BL21 strain cells by heat shock. After an overnight preculture in LB medium supplemented in ampicillin (50 μg/ml) and chloramphenicol (35 μg/ml), bacteria were diluted (1:1,000) into 250‐ml LB medium and grown at 37°C with agitation. When the culture OD_600nm_ reached 0.8, protein production was induced by the addition of isopropyl β‐D‐1‐thiogalactopyranoside and the culture was grown at 18°C overnight with agitation. Bacteria were pelleted, resuspended in NPI10 buffer (50‐mM NaH_2_PO_4_, 300‐mM NaCl, 10‐mM imidazole, pH 8) and sonicated (Vibracell 75455; Bioblock Scientific). After centrifugation, the 6His‐tagged p53 protein was purified from clarified lysate using Protino® Ni‐NTA agarose kit (Macherey‐Nagel): 1 ml of Ni NTA agarose beads was added and incubated overnight, at 4°C under continuous rotation. Beads were washed three times in NPI20 buffer (50‐mM NaH_2_PO_4_, 300‐mM NaCl, 20‐mM imidazole, pH 8). The 6His‐tagged p53 protein was finally eluted in NPI250 (50‐mM NaH_2_PO_4_, 300‐mM NaCl, 250‐mM imidazole, pH 8) and dialysed against 10‐mM PBS, 70‐mM NaCl, pH 7.5 overnight at 4°C.

### 4.11 Pull down assay

An in vitro pull‐down approach was used to find interactions between p53 and EtROP1. Recombinant EtROP1, purified from transiently transfected HEK293T, was mixed with 6His‐tagged p53 retained on Ni NTA agarose beads (Macherey‐Nagel) and incubated overnight, at 4°C under continuous rotation. Beads were washed three times in NPI20 buffer (50‐mM NaH_2_PO_4_, 300‐mM NaCl, 20‐mM imidazole, pH 8). Specifically bound proteins were eluted in 30 μl of NPI250 (50‐mM NaH_2_PO_4_, 300‐mM NaCl, 250‐mM imidazole, pH 8) and analysed on Coomassie‐stained SDS‐PAGE gels and immunoblots using an anti‐FLAG antibody (F7425, Sigma) to reveal the retained EtROP1 forms.

### 4.12 Annexin V assay

Two days posttransfection, HEK293T cell apoptosis was induced by addition of 2.5‐μg/ml actinomycin D (Sigma; for 24 or 48 hr). After apoptosis induction, cells were trypsinised, pooled, and centrifuged. HEK293T cells and epithelial cells from the caecum were incubated with Annexin V‐ PE (ImmunoTools) to determine the apoptosis rate, in Annexin binding buffer (10‐mM HEPES pH 7.4, 140‐mM NaCl, 2.5‐mM CaCl_2_) and with fixable viability dye efluor780 (Affymetrix eBioscience) in order to determine the mortality rate. Cells were preserved on ice until flow cytometry analysis on a MoFlo Astrios EQ (Beckman Coulter).

### 4.13 Caspase 3/7 activity assay

One day posttransfection, cells apoptosis was induced by 2.5‐μg/ml actinomycin D for 24 hr. Then, GFP‐positive and mCherry‐positive cells (i.e., transfected cells and infected cells) and GFP‐negative and mCherry‐negative cells (i.e., nontransfected cells and noninfected cells) were separately sorted by flow cytometry on a MoFlo Astrios EQ (Beckman Coulter) high‐speed cell sorter and distributed into 96‐well plates (10^4^ or 4 × 10^4^ cells/well). Caspase 3/7 activity was measured using the caspase‐Glo assay kit (Promega). Briefly, the caspase‐Glo reagent was added to each well (100 μl), and the plate was mixed gently and then incubated at room temperature for 1.5 hr. The luminescence of each sample was measured in a Glomax multidetection system photometer (Promega). The experiments were performed in triplicate.

### 4.14 Cell cycle assay

Transient transfected cells were grown for 2 days, fixed in ethanol, and frozen for 2 hr at −20°C. Then, cells were permeabilised with 0.25% Triton‐X‐100 and treated with RNases (0.2 mg/ml, Sigma), stained with propidium iodide (Molecular Probes, 5 μg/ml) and analysed by flow cytometry (MoFlo Astrios EQ Beckman Coulter). The percentage of cells in each cell cycle phase was assessed by using the MultiCycle software for Windows (Phoenix flow system, Inc.). At least 10,000 cells were collected for each condition, in three independent experiments.

### 4.15 Quantitative reverse transcription PCR

Total RNA from GFP‐positive and mCherry‐positive cells (i.e., transfected cells and infected cells) and GFP‐negative and mCherry‐negative cells (i.e., nontransfected cells and noninfected cells) was purified using the Nucleopsin XS kit (Macherey‐Nagel). RNA were extracted as described in the manufacturer's manual and suspended in nuclease‐free water. The Superscript™ II First Strand Synthesis System (Invitrogen), with oligo (dT)15 primer (Promega), was used to synthetise cDNA. Quantitative RT‐PCR was carried out using the IQ™ SYBR Green Supermix (Qiagen). The qPCR was performed using the following protocol: 95°C for 3 min and 40 cycles at 95°C for 10 s and 60°C for 30 s followed by 65°C for 30 s. The melting curve was generated at 65°C for 5 s followed by gradual heating (0.5°C/s) to 95°C. BAX, BCL_2_, and P21 gene expression values were normalised to the human housekeeping β‐actin (actin) and glyceraldehyde 3‐phosphate dehydrogenase (GAPDH) transcripts and the avian housekeeping β‐actin, G10 and GAPDH transcripts (see primers in Table S3). The relative gene expression levels were determined using the 2^(−Δct)^ method, and each experiment was performed in triplicate.

### 4.16 Production and purification of a recombinant EtROP1‐FLAG protein

Prior to in vitro kinase assays, various forms of a recombinant EtROP1‐FLAG were produced in eukaryotic system and purified using anti‐FLAG affinity chromatography. Briefly, cells transiently expressing the wt, the mutated or truncated forms of the EtROP1 protein were lysed in lysis buffer (50‐mM Tris–HCl pH 7.4; 150‐mM NaCl, 1‐mM EDTA, and 1% Triton‐X100) containing protease inhibitors. Cleared lysates were incubated with an anti‐FLAG‐M2‐affinity gel (Sigma‐Aldrich) under continuous rotation. After several washes (50‐mM Tris–HCl, pH 7.4; 150‐mM NaCl), immunoprecipitated proteins were eluted by competition using a 3X FLAG peptide (Sigma‐Aldrich).

### 4.17 Kinase activity assay

An in vitro kinase activity assay was performed to assess the phosphorylation activity of EtROP1. Kinase assay reactions were performed using 1 μg of purified EtROP1 (WT, mutated or truncated). Recombinant proteins were incubated with heterologous substrates (casein or Myelin Basic Protein, MBP) or crude cell lysate at 37°C for 30 min in a kinase buffer (60‐mM Tris–HCl, pH 7.5; 60‐mM MgCl_2_; 6‐mM MnCl_2_, NaF 0.1 M, glycerophosphate 0.3 M) supplemented with 5‐μM unlabelled ATP and 10‐μCi [γ‐^32^P]ATP (Perkin‐Elmer). After incubation, the reaction was stopped by addition of Laemmli buffer and heat denaturation at 95°C for 5 min. Phosphorylated proteins were separated on 10% SDS‐PAGE gels. The gels were stained with Coomassie Brilliant Blue and placed in a gel dryer (Bio‐Rad), exposed to an X‐ray film in an autoradiography cassette (Bio‐Rad), and visualised by autoradiography.

### 4.18 Western blot analysis

Total protein extracts from free parasites or from cells were prepared using a complete protease inhibitor cocktail (Roche) in the lysis buffer. Extracts were clarified by centrifugation and the protein concentration in the supernatant was measured using the DC Protein Assay Kit (Bio‐Rad). Protein samples were mixed with a denaturing sample buffer, boiled for 5 min and resolved by SDS‐PAGE. Proteins were transferred onto PVDF membranes (Bio‐Rad). Membranes were blocked by incubation in TBST (20‐mM Tris, 150‐mM NaCl, 0.1% (*v*/v) Tween 20) containing 5% nonfat dry milk and incubated with the appropriate primary antibodies. Anti‐p21 (#1026, Cell Signalling technology, 1:250) and Anti‐p53 (sc‐99, Santa Cruz, 1:100) were used. Β‐actin level (#4970, Cell Signalling technology, 1:1 000) was used as loading control. Detection was performed with HRP‐conjugated secondary antibodies (Pierce‐Thermo Scientific, 1:1,000). Band intensity was quantified by ImageJ software (Rasband, 1997). Each band was selected, circumscribed with rectangular selection using “Gels” function, followed by quantification of peak area of obtained histograms. Data were acquired as arbitrary area values.

### 4.19 Immunofluorescence assay

HFF monolayers were grown on coverslips and infected with *T. gondii* tachyzoites 24 hr before fixation with paraformaldehyde 4%. After permeabilisation, parasites were stained with an anti‐FLAG or an anti‐TgROP2 (generous gift from J.‐F. Dubremetz) antibody. Staining was revealed by appropriate Alexa488‐ or Alexa594‐conjugated secondary antibodies (Invitrogen). Coverslips were mounted on slides using Vectashield medium with DAPI (Clinisciences). HEK293T cells infected by recombinant E. tenella mCherry strain were fixed. After permeabilisation, phosphorylation of p53 on residue Thr^387^ was detected with a rabbit polyclonal anti‐p53‐pThr^387^ antibody (Ab111543, Abcam). Images were acquired on a fluorescence microscope (Zeiss Axiovert 200 microscope) and processed with ImageJ for numeration analysis.

### 4.20 Statistical analyses

Statistical analyses were performed using Prism GraphPad. The quantitative results were determined by one‐way analysis of variance and two‐way analysis of variance. The results were expressed as means *±* SEM. Statistical significance levels were considered as follows: *p* value <0.05 (*), *p* value <0.01 (**), and *p* value <0.001 (***).

### 4.21 Ethics approval

Experimental protocols were performed in accordance with the French legislation (Décret: 2001‐464 29/05/01), the EEC regulation (86/609/CEE) about laboratory animals, and after authorisation by the local ethics committee for animal experimentation (CEEA VdL n°19): 2012‐11‐09 that was renewed on the 2016‐03‐04 (APAFIS N°2828).

## Supporting information

Table S1.
Supporting information

Table S2.
Supporting information

Table S3.
**Primers used for plasmids construction and RT‐qPCR**. Primers for human or chicken targets are indicated with Hu or Ch, respectively, in names.

Fig S1.
Alignment of ETH_00005190 and TgME49‐ROP17 amino acid sequences.
The identical and similar residues are highlighted in red and blue, respectively. The predicted signal sequences is highlighted by a blue box. The SΦXE motif, putative maturation site of the pro‐region, is highlighted by a grey box.
**Figure S2. Characterisation of the Toxoplasma gondii recombinant strain (Tg‐EtROP1‐FLAG**).
PCR analysis with primers specific for the N‐terminal part of EtROP1 using genomic DNA purified from of wild‐type or EtROP1‐FLAG‐transfected T. gondii ME49 as templates. The expected size is 703 bp. MW, molecular weight.
Figure S3. EtROP1 inhibits apoptosis in avian cells.
A. Caspase 3/7 activity in CLEC‐213 cells transfected with EtROP1‐GFP expression plasmids (wt and dead forms) or the control plasmid pcDNA‐GFP. Two days posttransfection, GFP positive cells (transfected cells) were flow cytometry sorted and the caspase activity measured using the fluorogenic z‐DEVD caspase 3/7 substrate and a Glomax photometer. ANOVA analysis was significant (p < .0001). Different letters refer to different statistical groups.
B. Bax/Bcl_2_ gene expression quantified by RT‐qPCR in CLEC‐213 cells transfected with EtROP1‐GFP expression plasmids (wt and dead forms) or the control plasmid pcDNA‐GFP. Two days posttransfection, GFP positive cells (transfected cells) were flow cytometry sorted for subsequent total RNA purification. Gene expression values were normalised to the avian housekeeping β‐actin, G10 and GAPDH transcripts. Values are expressed as fold increase versus non transfected cells. Different means between pairs of sample groups were analysed by a one‐way ANOVA.
Figure S4. EtROP1 induces G0/G1 cell cycle arrest in avian cells.
A. EtROP1 induces LMH cell cycle arrest in G1 phase.
Cell cycle distribution of LMH cells transfected with EtROP1‐GFP expression plasmids (wt and dead forms) or the control plasmid pcDNA‐GFP. Two days posttransfection, GFP positive cells (transfected cells) were flow cytometry sorted using iodide‐efluor780 staining to assess the percentage of cells in each phase (G0/G1, S, G2/M). Data represent the average from three independent experiments. Differences in cell cycle phases between sample groups were analysed by a chi‐squared test. Different letters refer to different statistical groups.
B. P21 gene expression quantified by RT‐qPCR in CLEC‐213 cells transfected with EtROP1‐GFP expression plasmids (wt and dead forms) or the control plasmid pcDNA‐GFP. Two days posttransfection, GFP positive cells (transfected cells) were flow cytometry sorted for subsequent total RNA purification. Gene expression values were normalised to the avian housekeeping β‐actin, G10 and GAPDH transcripts. Values are expressed as fold increase versus pcDNA‐GFP transfected cells. Different means between pairs of sample groups were analysed by a one‐way ANOVA. Different letters refer to different statistical groups.
C. P21 gene expression quantified by RT‐qPCR in epithelial cells from caeca infected with mCherry E. tenella recombinant strain. Eighty‐four hours postinfection, mCherry positive (infected cells) and negative (not infected) cells were flow cytometry sorted for subsequent total RNA purification. Analysis was run as in S4 B legend.
