# Supplementary material for: Eimeria tenella ROP kinase EtROP1 induces G0/G1 cell cycle arrest and inhibits host cell apoptosis
Source: Cell Microbiol. 2019 Apr 24;21(7):e13027. doi: 10.1111/cmi.13027 (PMC6593979; doi:10.1111/cmi.13027)
Supplement: Supplementary file 3 — Table S3. Primers used for plasmids construction and RT‐qPCR. Primers for human or chicken targets are indicated with Hu or Ch, respectively, in names. [file CMI-21-na-s003.docx]

**S3 Table. Primers used for plasmids construction and RT-qPCR**. Primers for human or chicken targets are indicated with Hu or Ch, respectively, in names.

| Primer |  | Sequence (5’ … 3’) |
| --- | --- | --- |
| Plasmid construction | | |
| TgROP1 promotor | F | TCCGTGAGCACGTTTGCTGAGAAGTTAG |
|  | R | CTAAACTGAGTTAGTTTCAGACGAGTG |
| pGEX-Cdom | F | GGGGATCCACCTTTGACTGTGTGGGCTACG |
|  | R | GGGTCGACTTAGCATTACTGGGTGTGATG |
| pET-p53 | F | GGGCCCCATATGATGGAGGAGCCGCAGTCAGATC |
|  | R | GACAGAAGGGCCTGACTCAGACTGAGGATCCGGACCT |
| pcDNA-ROP1-HA-FLAG | F | GGGGATCCACCATGCTTCGGCGCGGTGCCCTGCTCG |
|  | R | GGACCTGCGGCCGCTTACTTGTCGTCATCGTCTTTGTAGTCCATTACTGGGT GTGATGCA |
| pcDNA-ROP1-6His | F | GGGGATCCACCATGGATGCGTGCTTTGCTCGCACG |
|  | R | GGGCGGCCGCGAACTGAGTTAGTTTCAGACGAGTG |
| pcDNA-ROP1-Cdom-FLAG | F | GGGGATCCACCATGCTTCGGCGCGGTGCCCTGC |
|  | R | GGGCGGCCGCTTACTTGTCGTCATCGTCTTTGTAGTCGCATTACTGGGTGTGATG |
| pcDNA-ROP1-ΔCter-FLAG | F | GGGGATCCACCATGCTTCGGCGCGGTGCCCTGCTCG |
|  | R | GGACCTGCGGCCGCTTAGCATTACTGGGTGTGATG |
| pcDNA-ROP1-ΔNter-FLAG | F | GGGCCCGGATCCACCATGACCCTTCGGCGCGGTGCCCTGC |
|  | R | GGACCTGCGGCCGCTTACTTGTCGTCATCGTCTTTGTAGTCCATTACTGGGTGTGATGCA |
| pcDNA-ROP1-wt-GFP-FLAG | F | GGGGATCCACCATGCTTCGGCGCGGTGCCCTGCTCG |
|  | R | AGGTCCCTCGAGTTACTTGTCGTCATCGTCTTTGTAGTCCTTGTACAGCTCGTCCATGC |
| pcDNA-ROP1-ΔNter-GFP-FLAG | F | GGGCCCGGATCCACCATGACCTTTGACTGTGTGGGCTACG |
|  | R | AGGTCCCTCGAGTTACTTGTCGTCATCGTCTTTGTAGTCCTTGTACAGCTCGTCCATGC |
| pcDNA-ROP1-dead1-GFP-FLAG (K285A) |  | GGTGCTCAGTACGCCGGCGCGATCTATGCC |
| pcDNA-ROP1-dead2-GFP-FLAG (D404A) |  | CTGGTGCATTTCGCTATCAAGCCCCA |
| pcDNA-ROP1-NTE-FLAG | F | GGGCCCGGATCCACCATGGAGCAAGCCGGCAACTACAAC |
|  | R | AGGTCCCTCGAGTCACTTGTCGTCATCGTCTTTGTAGTCAACTATGCTCTGGTCGTAGC |
| pcDNA-ROP1-(aa151-330)-FLAG | F | GGGCCCGGATCCACCATGGGAAAGGGAACAAGCAGCCCC |
|  | R | AGGTCCCTCGAGTCACTTGTCGTCATCGTCTTTGTAGTCACAGAGAGGAAGCGAAAGATG |
| pcDNA-ROP1-(aa82-257)-FLAG | F | GGGCCCGGATCCACCATGGAGCAAGCCGGCAACTACAAC |
|  | R | AGGTCCCTCGAGTCACTTGTCGTCATCGTCTTTGTAGTCACAGAGAGGAAGCGAAAGATG |
| RT-qPCR | | |
| Hu_actine | F | TGGTGATGGAGGAGGTTTAGTAAGT |
|  | R | AACCAATAAAACCTACTCCTCCCTTAA |
| Hu_GAPDH | F | TCGACAGTCAGCCGCATCTTCTTT |
|  | R | GCCCAATACGACCAAATCCGTTGA |
| Hu_Bax | F | CATGGAGCTGCAGAGGATGAT |
|  | R | TTGCCGTCAGAAAACATGTCA |
| Hu_Bcl2 | F | TTGTGGCCTTCTTTGAGTTCGGTG |
|  | R | ACTCACATCACCAAGTGCACCTAC |
| Hu_P21 | F | GTCACTGTCTTGTACCCTTGTG |
|  | R | GGCGTTTGGAGTGGTAGAAA |
| Ch_actine | F | TGCTGTGTTCCCATCTATCGTG |
|  | R | AGTTGGTGACAATACCGTGTTCA |
| Ch_GAPDH | F | GTCCTCTCTGGCAAAGTCCAAG |
|  | R | CCACAACATACTCAGCAGCTGC |
| Ch_G10 | F | TCAAGGAAGGGTACGCTGACA |
|  | R | AACAGCCTCTGCATCCACAGT |
| Ch_Bax | F | TCCATTCAGGTTCTCTTGAC |
|  | R | ATAGCCAAACATCCAAACACAGA |
| Ch_Bcl2 | F | GATGACCGAGTACCTGAACC |
|  | R | CAGGAGAAATCGAACAAAGGC |
| Ch_P21 | F | GTCGGTTCTCCCCAGCG |
|  | R | GCAGTCACAGCTTGGGCTTA |
