# Supplementary material for: Eimeria tenella ROP kinase EtROP1 induces G0/G1 cell cycle arrest and inhibits host cell apoptosis
Source: Cell Microbiol. 2019 Apr 24;21(7):e13027. doi: 10.1111/cmi.13027 (PMC6593979; doi:10.1111/cmi.13027)
Supplement: Supplementary file 4 — Fig S1. Alignment of ETH_00005190 and TgME49‐ROP17 amino acid sequences. The identical and similar residues are highlighted in red and blue, respectively. The predicted signal sequences is highlighted by a blue box. The SΦXE motif, putative maturation site of the pro‐region, is highlighted by a grey box. Figure S2. Characterisation of the Toxoplasma gondii recombinant strain (Tg‐EtROP1‐FLAG). PCR analysis with primers specific for the N‐terminal part of EtROP1 using genomic DNA purified from of wild‐type or EtROP1‐FLAG‐transfected T. gondii ME49 as templates. The expected size is 703 bp. MW, molecular weight. Figure S3. EtROP1 inhibits apoptosis in avian cells. A. Caspase 3/7 activity in CLEC‐213 cells transfected with EtROP1‐GFP expression plasmids (wt and dead forms) or the control plasmid pcDNA‐GFP. Two days posttransfection, GFP positive cells (transfected cells) were flow cytometry sorted and the caspase activity measured using the fluorogenic z‐DEVD caspase 3/7 substrate and a Glomax photometer. ANOVA analysis was significant (p < .0001). Different letters refer to different statistical groups. B. Bax/Bcl2 gene expression quantified by RT‐qPCR in CLEC‐213 cells transfected with EtROP1‐GFP expression plasmids (wt and dead forms) or the control plasmid pcDNA‐GFP. Two days posttransfection, GFP positive cells (transfected cells) were flow cytometry sorted for subsequent total RNA purification. Gene expression values were normalised to the avian housekeeping β‐actin, G10 and GAPDH transcripts. Values are expressed as fold increase versus non transfected cells. Different means between pairs of sample groups were analysed by a one‐way ANOVA. Figure S4. EtROP1 induces G0/G1 cell cycle arrest in avian cells. A. EtROP1 induces LMH cell cycle arrest in G1 phase. Cell cycle distribution of LMH cells transfected with EtROP1‐GFP expression plasmids (wt and dead forms) or the control plasmid pcDNA‐GFP. Two days posttransfection, GFP positive cells (transfected cells) were f [file CMI-21-na-s004.pptx]

## Slide 1
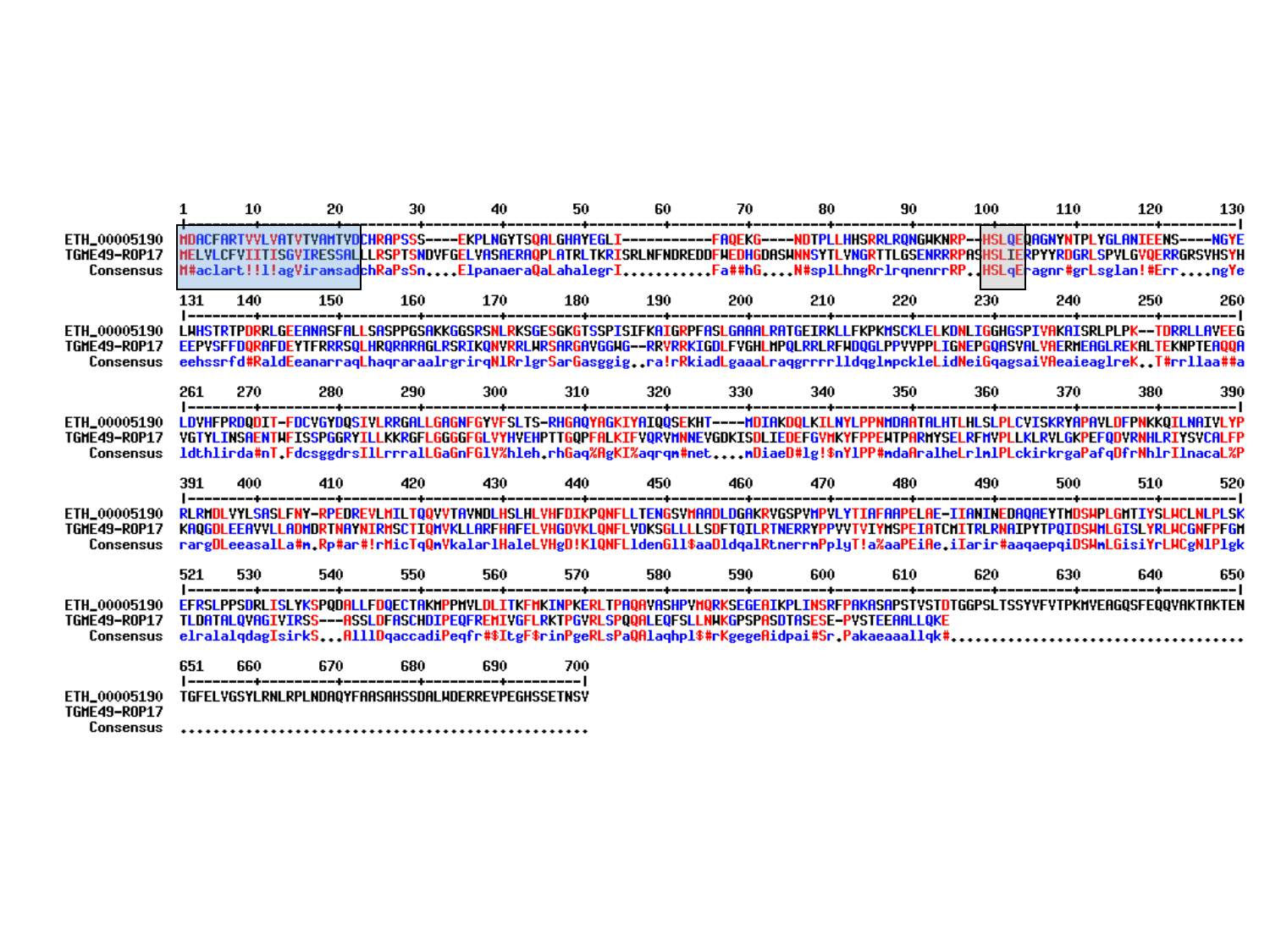

## Slide 2
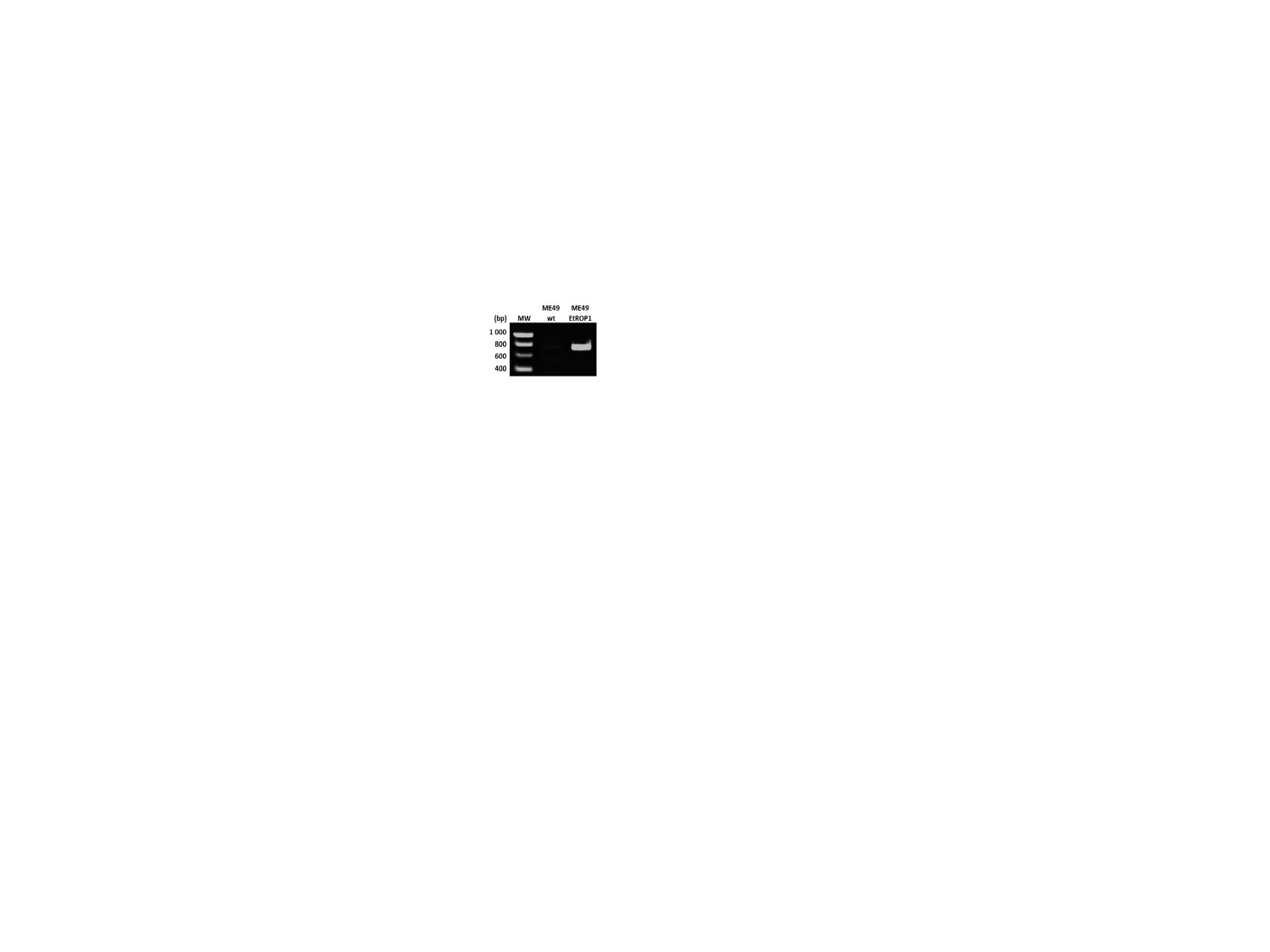

## Slide 3
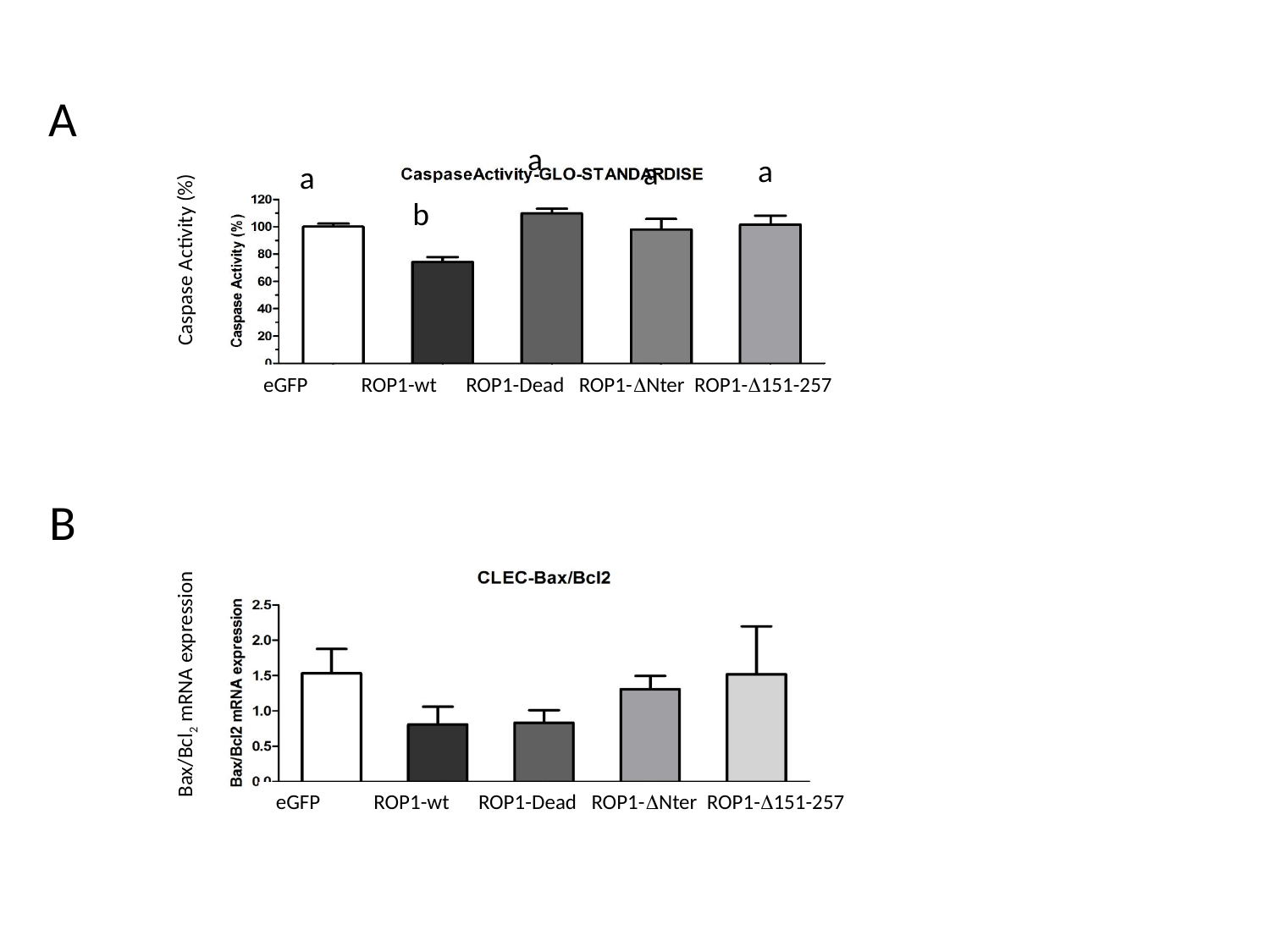

A
a
a
a
a
b
Caspase Activity (%)
 eGFP ROP1-wt ROP1-Dead ROP1-DNter ROP1-D151-257
B
Bax/Bcl2 mRNA expression
 eGFP ROP1-wt ROP1-Dead ROP1-DNter ROP1-D151-257

## Slide 4
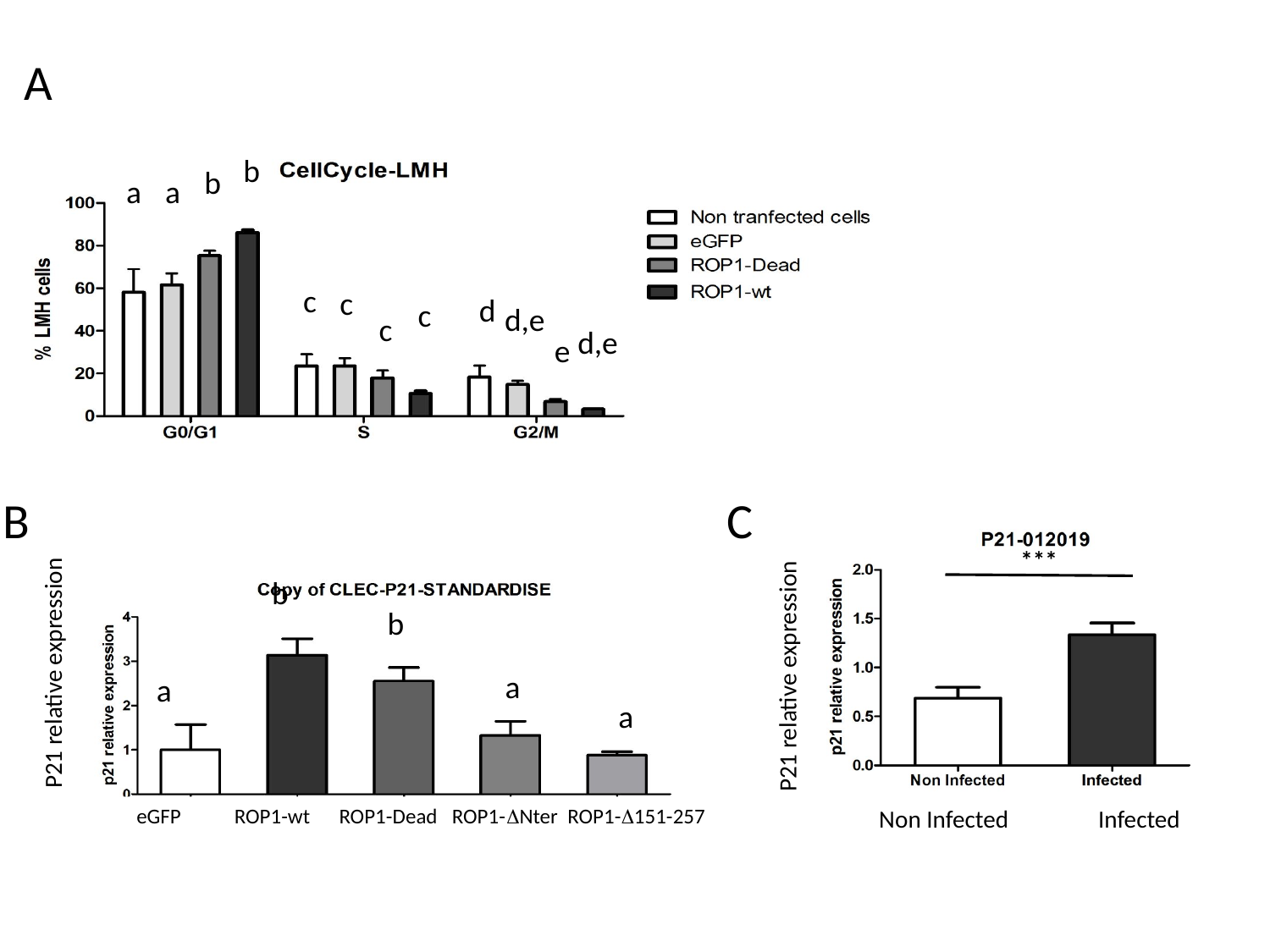

A
b
b
a
a
c
c
d
c
d,e
c
d,e
e
B
C
***
b
b
P21 relative expression
P21 relative expression
a
a
a
 eGFP ROP1-wt ROP1-Dead ROP1-DNter ROP1-D151-257
Non Infected
Infected
